# MzPIP2;1: An Aquaporin Involved in Radial Water Movement in Both Water Uptake and Transportation, Altered the Drought and Salt Tolerance of Transgenic *Arabidopsis*

**DOI:** 10.1371/journal.pone.0142446

**Published:** 2015-11-12

**Authors:** Lin Wang, Qingtian Li, Qiong Lei, Chao Feng, Yinan Gao, Xiaodong Zheng, Yu Zhao, Zhi Wang, Jin Kong

**Affiliations:** 1 College of Horticultural Plants, China Agricultural University, Beijing, China; 2 College of Biological Sciences, China Agricultural University, Beijing, China; Key Laboratory of Horticultural Plant Biology (MOE), CHINA

## Abstract

**Background:**

Plants are unavoidably subjected to various abiotic stressors, including high salinity, drought and low temperature, which results in water deficit and even death. Water uptake and transportation play a critical role in response to these stresses. Many aquaporin proteins, localized at different tissues, function in various transmembrane water movements. We targeted at the key aquaporin in charge of both water uptake in roots and radial water transportation from vascular tissues through the whole plant.

**Results:**

The *MzPIP2;1* gene encoding a plasma membrane intrinsic protein was cloned from salt-tolerant apple rootstock *Malus zumi* Mats. The *GUS* gene was driven by *MzPIP2;1* promoter in transgenic *Arabidopsis*. It indicated that MzPIP2;1 might function in the epidermal and vascular cells of roots, parenchyma cells around vessels through the stems and vascular tissues of leaves. The ectopically expressed *MzPIP2;1* conferred the transgenic *Arabidopsis* plants enhanced tolerance to slight salt and drought stresses, but sensitive to moderate salt stress, which was indicated by root length, lateral root number, fresh weight and K^+^/Na^+^ ratio. In addition, the possible key *cis*-elements in response to salt, drought and cold stresses were isolated by the promoter deletion experiment.

**Conclusion:**

The MzPIP2;1 protein, as a PIP2 aquaporins subgroup member, involved in radial water movement, controls water absorption and usage efficiency and alters transgenic plants drought and salt tolerance.

## Introduction

Plants are unavoidably subjected to various abiotic stressors, including high salinity, drought and low temperature. These stressors result in water deficit, wilt and even death in severe cases [1–3]. The water uptake from soil to root epidermal cells and the radial water transportation from vessels to parenchyma cells are the critical steps limiting the efficiency of water uptake and usage, which are especially important under water deficit [4]. Both of these two steps completely depend on transmembrane water transportation, in which aquaporins play an essential role.

Aquaporins are membrane proteins, which belong to the major intrinsic protein (MIP) family, involved in transmembrane water movement. Their activity to transport water across membrane was proved by *in vivo* and *in vitro* experiments. *In vitro*, maize ZmPIP2a was able to transport large amounts of water in the *Xenopus* ovocytes system [5]. *In vivo*, the lily *PIP1* increased osmotic water permeability coefficient (P_*f*_) in transgenic tobacco leaf protoplasts [6]. In *Arabidopsis PIP* mutant, P_*f*_ of leaves protoplasts decreased markedly, and in the *PIP*-antisense transgenic *Arabidopsis*, the L_*p*_ (i.e. hydraulic conductivity) of cells went down as well [7,8].

As the key proteins in plant growth and development, abundant aquaporins exist in the most living organisms (archaea, eubacteria, fungi, plants and animals), involved in many physiological processes including water uptake and transportation, reproduction and photosynthesis [9–11]. They are divided into five groups according to the sequence similarity: the plasma membrane intrinsic protein (PIPs), the tonoplast intrinsic protein (TIPs), the Nodulin26-like intrinsic protein (NIPs), the small basic intrinsic protein (SIPs) and the X intrinsic protein (XIPs) [12–15]. They have been reported to be involved in hydraulic conductance in roots, stems and leaves, cooperating water uptake and transportation in the whole plant [8,16,17].

Aquaporins play especially vital roles in response to water deficit. Plants evolved a sophisticated mechanism to fine-tune the abundance, relocation and gating of aquaporins, which facilitate plants to modify water permeability rapidly and reversibly [18–22]. The abundance of the aquaporin proteins in the membranes are regulated at transcriptional and post-translational levels. Many aquaporin genes changed their expression under water deficit [23–25]. For the post-translational strategy, the translated aquaporin proteins were re-localized or endocytosed through the internalization of clathrin-coated vesicles of raft-associated domains to rapidly change their levels in the target membranes under water deficit [26–30]. In addition, the activity of the aquaporin proteins was also manipulated by the dephosphorylation and heteromerization of PIPs, which controls the gating for water movement [4].

Because of the variant roles of aquaporins in response to water deficit, the transgenic plants overexpressing aquaporin genes exhibited different phenotypes under stresses. *PgTIP1* gene conferred transgenic *Arabidopsis* enhanced salt tolerance [31]. But *HvPIP2;1* made the transgenic rice more sensitive to salt stress [32]. Likewise, transgenic *Arabidopsis* ectopically expressing *VfPIP1* showed enhanced drought tolerance [21]. But transgenic plants with increased *PIP1b* and *HvPIP2;1* expression were sensitive to drought stress [32,33]. Although aquaporins have been intensively investigated in model plants, there are a few systematic researches on their roles in response to different stresses including salt, drought and cold stresses. Most importantly, there are few reports on the aquaporin playing key roles in both water uptake and transportation in response to water deficit.

In this work, we cloned *MzPIP2;1* gene encoding plasma membrane intrinsic protein from a salt-tolerant apple rootstock *Malus zumi* Mats. The transverse and longitude paraffin sections profiled the localization of *MzPIP2;1*, suggesting it might be the key aquaporin involved in water movement from soil to root epidermal cells and from vessels to parenchyma cells all through the whole plant. The phenotype of the transgenic *Arabidopsis* ectopically expressed *MzPIP2;1* and the *GUS* expression driven by different regions of the *MzPIP2;1* promoter were observed under salt, drought and cold stresses. These results systematically confirmed the crucial roles of MzPIP2;1 in both water uptake and radial water transportation, which was concluded to control the efficiency of water absorption and usage under water deficit. We isolated the possible stress-response *cis*-elements. It laid a foundation for isolating the genes regulating aquaporins, which remains unclear.

## Materials and Methods

### Plant materials and growth conditions

The *Arabidopsis* (*Arabidopsis thaliana* ecotype Colombia 0) seeds were sterilized with 1% sodium hypochlorite for 12min, 75% alcohol for 30sec, and washed 4 times with sterile water. After vernalized 3 days in dark at 4°C, the seeds were sowed and grown vertically on Murashige and Skoog (MS) medium in a growth chamber of 16h light (~150 μEm^-2^ sec^-1^) /8h dark at 22±2°C. When the seedlings had four leaves, they were transplanted into the soil.

### Clone of *MzPIP2;1* gene and bioinformatics analysis

The salt-responsive *MzPIP2;1* gene was isolated by microarray, in which the 15,000 cDNA clones were from the cDNA library constructed by our lab [34]. Total RNA was extracted from the leaves of *Malus zumi* Mats (apple rootstock) under salt stress using the EASYspin Plant RNA Rapid Extraction Kit (Biomed, Beijing, China), and immediately used for cDNA synthesis with a first-strand cDNA synthesis kit (TaKaRa, Shiga, Japan). The full-length *MzPIP2;1* cDNA of 1094 bp was amplified using the 2×ES Taq MasterMix (CWBIO, Beijing, China) with the specific primers carrying the restriction sites *Bam*HI and *Xba*I (forward primer: 5’-GGATCCTCCCAAACTCTGTGAAGC-3’, reverse primer: 5’-TCTAGACACAAAGCATAAGTTAAGCAC-3’). The PCR was performed as follows: 94°C for 3min; 30 cycles at 94°C for 30sec, 52°C for 30sec, and 72°C for 50sec; and 72°C for 10min. The PCR product was cloned into the PMD^®^19-T simple vector (TaKaRa, Shiga, Japan) and sequenced. The NCBI accession number of *MzPIP2;1* was GenBank: KR349329.

The conserved domain of MzPIP2;1 was predicted on line at http://smart.embl-heidelberg.de/. Meanwhile, its molecular weight and isoelectric point were analyzed on line at http://www.bio-soft.net/sms/ and http://web.expasy.org/compute_pi/, respectively. The multiple alignments of MzPIP2;1 amino acid sequence were conducted using DNAMAN software to identify the shared domains among arabidopsis, rice, maize and horticultural plants including pear, grape, castor, spinach, cabbage, poplar and apple, using its homologous proteins: AtPIP2;1 (AEE79084.1), OsPIP2;1 (Q8H5N9.1), PcPIP2;2 (BAB40143.1), VvPIP2 (ABC84558.1), RcPIP2;1 (EEF29633.1), AtPIP3 (AAB36949.1), AtPIP2;5(AEE79295.1), AtPIP1;4(AEE81879.1), SoPIP1;2 (AAR23268.1), ZmPIP1;2 (NP_001104934.1), OsPIP1;3 (Q9SXF8.2), BoPIP3 (AAG30607.1), PcPIP1;1 (BAB40142.1), PcPIP2;1 (BAB40141.1), PtPIP2;5(CAH60724.1) and PtPIP2;2 (CAH60721.1). The phylogenetic tree was constructed using the neighbor-joining method and a bootstrap test with 1000 iterations by MEGA5.2 software.

### The subcellular localization of *MzPIP2;1* gene

The coding region of *MzPIP2;1* without stop codon was amplified by PCR using the primers with *Eco*RI/*Bam*HI restriction sites (forward primer: 5’- GAATTCATGGCCAAGGACATGGA -3’, reverse primer: 5’- GGATCCCAAACACTCGGGTGG -3’). The PCR cycles were performed as follows: 94°C for 3min; 30 cycles at 94°C for 30sec, 52°C for 30sec, and 72°C for 50sec; and 72°C for 10min. The PCR product was cloned into the PMD^®^19-T simple vector (TaKaRa, Shiga, Japan). After sequencing, it was digested with *Eco*RI/*Bam*HI and subcloned into transient expression vector pEZS-NL to be expressed as a fusion protein with an eGFP (enhanced Green Fluorescent Protein).

The protoplasts were extracted from the leaves of 3-week-old *Arabidopsis* grown in greenhouse. The *MzPIP2;1*-*eGFP* and *eGFP* control plasmids were introduced to protoplasts by PEG mediated transformation as described by Yoo *et al*. (2007)[35]. A total of 100μL protoplasts and 10μL *MzPIP2;1*-*eGFP* or *eGFP* control plasmid was mixed gently, after which 110μL PEG was added and incubated for 20min. After washing twice by W5 solution (154mmol/L NaCl, 125mmol/L CaCl_2_, 5mmol/L KCl, 2mmol/L MES, pH5.7), the protoplasts were diluted by 1mL W5 solution, and incubated for 16–24h at 23°C in culture plate under low light conditions. The protoplast solution was observed using confocal microscopy (Nikon, Japan) with 600× magnification (objective magnification×eyepiece magnification = 60×10).

### Clone of *MzPIP2;1* promoter and deletion experiment

According to the instructions of TaKaRa LA PCR^™^ *in vitro* Cloning Kit, the chromosome walking of *MzPIP2;1* was conducted. The DNA of *Malus zumi* Mats was digested completely with restriction enzymes *Xba*I, *Bam*HI and *Eco*RI, and ligated with adapters carrying restriction sites *Xba*I, *Bam*HI and *Eco*RI. The first PCR reaction was conducted using the adapter primer C1 and the gene specific primer PGSP1 designed by the cDNA sequence of *MzPIP2;1*. The PCR cycles were performed as follows: 94°C for 3min; 35 cycles at 94°C for 30sec, 62°C for 30sec, and 72°C for 2min; and 72°C for 10min. Then PCR product was diluted 100 times and used for template, which was amplified with inner adapter primer C2 and the *mxIRT1* gene specific primer PGSP2 (94°C for 3min; 35 cycles at 94°C for 30sec, 56°C for 30sec, and 72°C for 2min; and 72°C for 10min). The PCR product was cloned into the PMD^®^19-T simple vector (TaKaRa, Shiga, Japan) and sequenced. The *MzPIP2;1* promoter sequences of 1500bp were blasted on Apple Genome Database (http://genomics.research.iasma.it/). Its specific forward and reverse primers, carrying restriction sites *Bam*HI and *Sma*I, were designed to amplify exactly 1500bp before ATG. The PCR was performed as follows: 94°C for 3min; 35 cycles at 94°C for 30sec, 55°C for 30sec, and 72°C for 2min; and 72°C for 10min. The PCR product was cloned into the PMD^®^19-T simple vector (TaKaRa, Shiga, Japan) and sequenced.

The *MzPIP2;1* promoter sequence was analyzed on line at website http://bioinformatics.psb.ugent.be/webtools/plantcare/html/. Based on *cis*-elements analysis of *MzPIP2;1* promoter, the primers were designed at different regions on the Primer Premier 5.0 software. The specific forward primers of two deletion promoters, carrying restriction site *Bam*HI, were designed on 948bp and 644bp before ATG, respectively. The PCR program was performed as follows: 94°C for 3min; 28cycles at 94°C for 30sec, 50°Cfor 30sec, and 72°C for 90sec; and 72°C for 10min. The PCR product was subcloned into the PMD^®^19-T simple vector (TaKaRa, Shiga, Japan) and sequenced. The sequences of all the primers were shown in Table 1.

**Table 1:** The sequences of primers in the *MzPIP2;1* promoter construction.

The name of primer	The sequences of primer
C1	5’-GTACATATTGTCGTTAGAACGCGTA ATACG ACTCA-3’
C2	5’-CGTTAGAACGCGTAATACGACTCACTATAG GGAGA-3’
PGSP1	5’-TGAAATCCCAGCAGTGCAGTAAACAAGG-3’
PGSP2	5’- ACAGTGACGTAGAGAAAGAGAAGAGTGG-3’
Pro_*MzPIP2;1*_-F	5’-GGATCCTCGTTCAAGAACCGCC-3’
Pro_*MzPIP2;1*_-R	5’-CCCGGGTAAAAAGGCTTAAGGTTCAC-3’
Pro_*MzPIP2;1*_-d1-F	5’-GGATCCTTCACTGTTTGATGCCATA-3’
Pro_*MzPIP2;1*_-d1-R	5’-CCCGGGTAAAAAGGCTTAAGGTTCAC-3’
Pro_*MzPIP2;1*_-d2-F	5’-GGATCCAATATTCATGCGTTCGACA-3’
Pro_*MzPIP2;1*_-d2-R	5’-CCCGGGTAAAAAGGCTTAAGGTTCAC-3’

### Ectopically expressing *MzPIP2;1* gene and *GUS* gene driven by *MzPIP2;1* promoter and two deletions in transgenic *Arabidopsis* plants

The *MzPIP2;1* gene was inserted into plant expression vector pCB302-3 with *Bam*HI/*Xba*I, driven by the cauliflower mosaic virus (CMV) 35S promoter. The full-length and two deletions of *MzPIP2;1* promoter were inserted into plant expression vector pBI121 with *Bam*HI/*Sma*I, respectively. The pCB302-3- Pro_*35S*_::MzPIP2;1 plasmid, pBI121-Pro_*MzPIP2;1*_::GUS plasmid, pBI121-Pro_*MzPIP2;1*_-d1::GUS and pBI121-Pro_*MzPIP2;1*_-d2::GUS plasmids were introduced into *Agrobacterium tumefaciens* strain GV3101 using the freeze-thaw method [36], respectively. Then they were introduced into Col-0 *Arabidopsis* plants using the floral dip method [37]. The T0 seeds were sowed on soil spraying 0.01% Basta or 1/2 Murashige-Skoog (MS) growth media containing 25mg/L kanamycin to screen the T1 generation transgenic plants ectopically expressing *MzPIP2;1* or *GUS* gene, respectively. Then the selected T1 seedlings were confirmed by PCR and transplanted into soil for seeds collection in a growth chamber at 22±2°C in a 16/8h light/dark cycles. The single-inserted T2 plants were judged by the 3:1 segregation ratio. The single-inserted homologous T3 transgenic lines were obtained when no segregation happened. The expression of foreign genes were detected by semi-quantitative RT-PCR using the *MzPIP2;1*-specific primers (forward primer: 5’-GTGCCAACTCACTTAGCGA-3’, reverse primer:5’-AATCGGGTTTGCTGTGTTCA -3’).

### Abiotic stress treatments and phenotype observation

The T3 seeds of three single-insertion lines and the wild-type were sterilized and sowed on MS medium. The 7-day-old seedlings were transferred to MS medium containing 100mM or 130mM NaCl for salt stress treatment, 300mM mannitol for drought stress in a growth chamber at 22±2°C. For cold stress treatment, the seedlings were placed at 4°C. The seedlings growing under normal conditions (short-day conditions (8/16h light/dark cycles) with a light intensity of ~150 μEm^-2^ sec^-1^) were used as control. The phenotypes were observed 2 weeks later after treatment. The root length, the lateral root number and the fresh weight of transgenic and wild-type plants under every treatment were recorded, respectively. In addition, the treated and wild-type *Arabidopsis* were harvested for the Na^+^ and K^+^ detection. Each experiment was repeated three times independently.

### The measurement of Na^+^ and K^+^ in transgenic and wild-type *Arabidopsis* plants

The HNO_3_-H_2_O_2_-microwave digestion method was applied for the measurement of Na^+^ and K^+^. After the plant materials were washed 2–3 times with water and another 2–3 times with deionized water, the samples were put into envelopes for drying 30min at 105°C and 48h at 70°C. The dried samples were grinded into powder with mortars and pestles. A total of 50mg samples were transferred to tubes and mixed with 6mL guarantee reagent HNO_3_, which were shaken gently overnight. Then 2 mL guarantee reagent H_2_O_2_ was add for a reaction of 10min. The tubes were put into microwave digestion instrument (CEM MARS, USA) for digestion with the program (120°C, 8min; 150°C, 9min; 185°C, 24min; cool down, 25min). After the tubes cooled down, the solutions were transferred and diluted to 25mL with distilled water. After they were kept still for 1h, the supernatants were moved into 10mL centrifuge tubes for measurement by atomic absorption spectrometer (Varian, USA). The 0, 5, 10, 20 and 50ppm Na^+^ and K^+^ were used as standard solution.

### *GUS* expression analysis driven by *MzPIP2;1* promoter and its deletions under abiotic stresses

The 10-day-old *Arabidopsis* seedlings expressing *GUS* gene driven by the *MzPIP2;1* full-length promoter and its two deletions were placed on filter paper soaking 150mM NaCl solution for salt stress treatment, 200mM mannitol solution for drought stress treatment, and deionized water as control. Except for the cold stress at 4°C, other treatments were conducted in a growth chamber with cycles of 8h light (~150 μEm^-2^ sec^-1^) and 16h dark at 22±2°C. After 24h treatment, the plants were sampled and vacuumized for 10min and incubated at 37°C overnight in the GUS solution (10mL solution: 3mL 0.2M NaH_2_PO_4_·H_2_O, 6mL 0.2M Na_2_HPO_4_·2H_2_O, 200μL 100mM X-Gluc stock, 1mL methyl alcohol). After decolorized in 70% ethanol at 37°C overnight, the samples were placed on plates carrying deionized water for observation by stereoscope with 7.3× magnification (objective magnification × eyepiece magnification = 0.73×10) (Leica, Germany).

Similar study in soil was conducted using 4-week-old seedlings. The transgenic and the wild-type plants were not watered in drought stress treatment, watered with 200mM NaCl for salt stress treatment and cultured at 4°C for cold stress treatment, respectively. After 24h treatment, the stems and roots of transgenic and wild-type plants were sampled for GUS analysis as described above. The experiments were repeated three times. In addition, to determine localization of MzPIP2;1 in the whole plant, paraffin sections of the transgenic *Arabidopsis* stems expressing the *GUS* gene driven by *MzPIP2;1* full-length promoter were made. The longitudinal section and transverse section of stems were observed by light microscope with 600× magnification (objective magnification × eyepiece magnification = 60×10) (Leica, Germany). In addition, the GUS expression in roots and leaves were also observed under light microscope 400× magnification (objective magnification × eyepiece magnification = 40×10).

### Statistical analysis

One-way ANOVA was used for statistical evaluations by SPSS software. The differences were significant when P<0.05 and very significant when P<0.01.

## Results

### MzPIP2;1 belongs to a PIP2 subfamily of aquaporins

**Fig 1:**
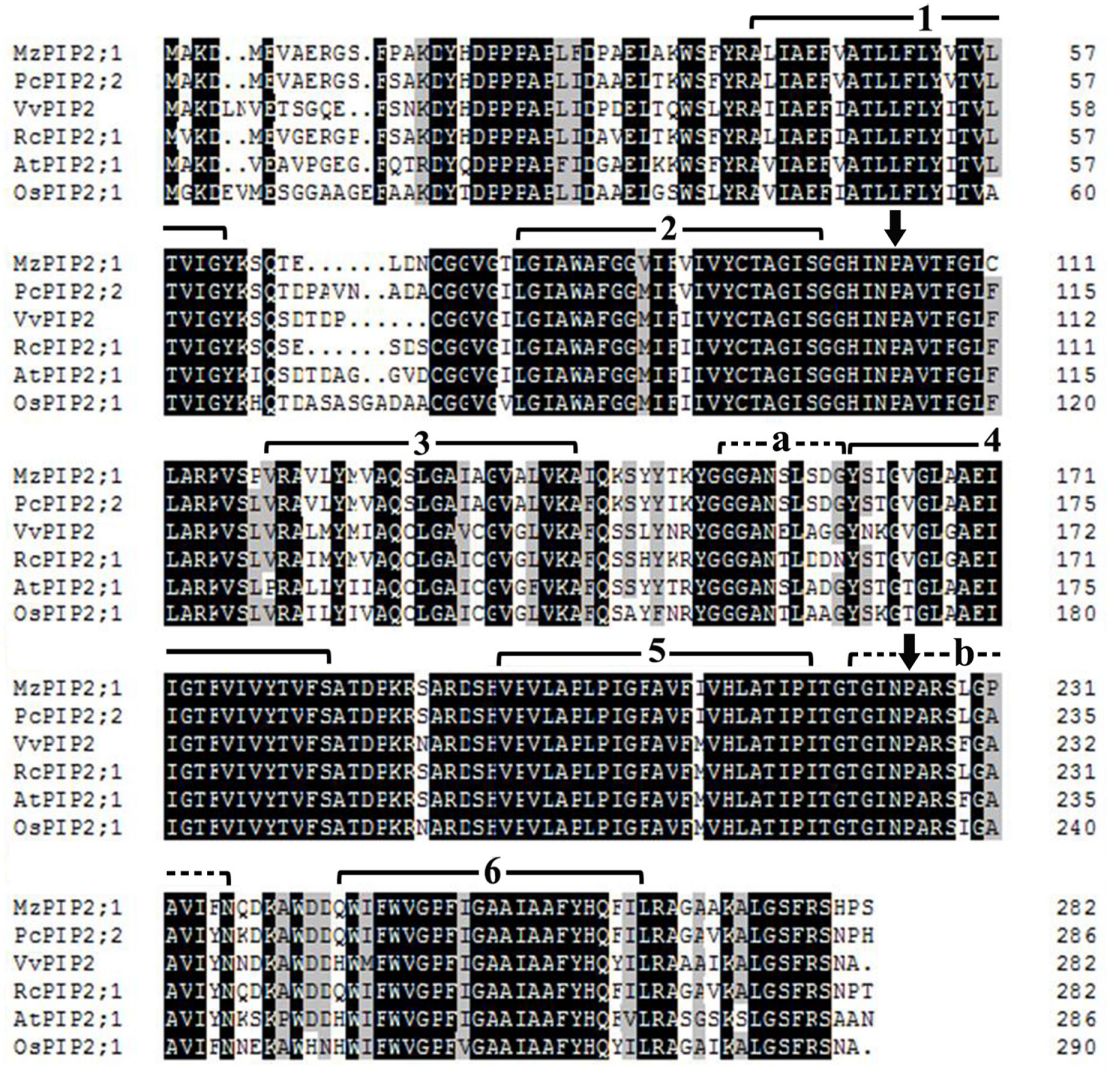
The multiple alignments of MzPIP2;1. The black color highlights the same amino acid sequence. The gray color identifies the sequence with only one amino acid difference. The solid lines with numbers, the arrows and the dash lines with lowercases show the transmembrane domains, the conserved NPA domains (Asn-Pro-Ala) and the signature sequences of plasma membrane water channel protein family, respectively.

**Fig 2:**
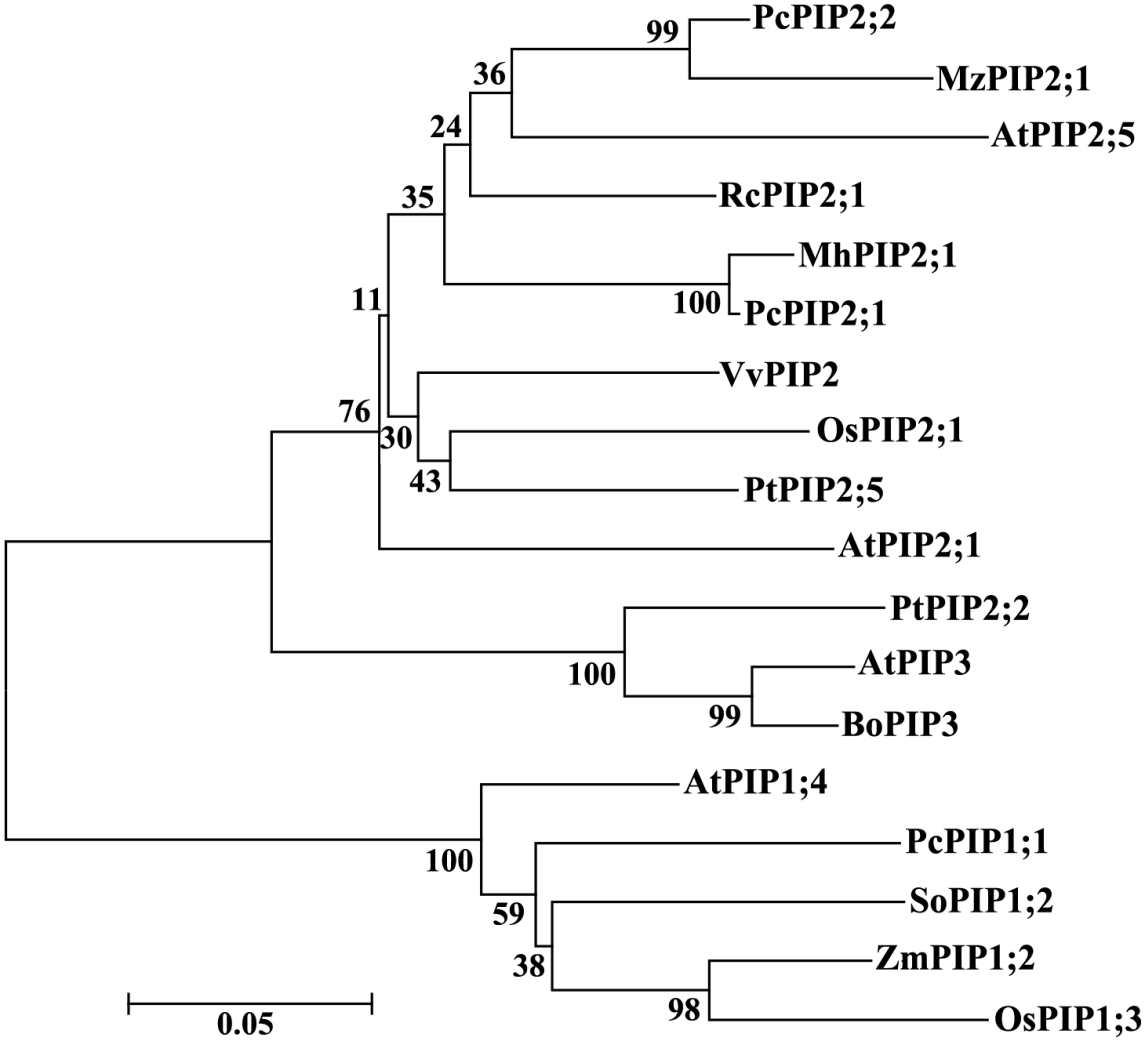
The phylogenetic tree of MzPIP2;1. The phylogenetic tree was constructed using the neighbor-joining method and a bootstrap test with 1000 iterations, using MEGA5.2 software. The GenBank accession numbers are AtPIP2;1 (AEE79084.1), OsPIP2;1 (Q8H5N9.1), PcPIP2;2 (BAB40143.1), VvPIP2 (ABC84558.1), RcPIP2;1(EEF29633.1), AtPIP3 (AAB36949.1), AtPIP2;5(AEE79295.1), AtPIP1;4(AEE81879.1), SoPIP1;2 (AAR23268.1), ZmPIP1;2 (NP_001104934.1), OsPIP1;3 (Q9SXF8.2), BoPIP3 (AAG30607.1), PcPIP1;1 (BAB40142.1), PcPIP2;1 (BAB40141.1), PtPIP2;5(CAH60724.1) and PtPIP2;2 (CAH60721.1).

The full-length cDNA of *MzPIP2;1* (GenBank: KR349329) was amplified from *Malus zumi*. It is 1094bp long including an open reading frame (ORF) of 852bp, a 5’-UTR of 65bp and a 3’-UTR of 177bp. The *MzPIP2;1* gene encodes 283 amino acids with predicted relative molecular mass of 30.08KD and predicted isoelectric point of 8.68. The multiple alignments indicated that MzPIP2;1 shared a high similarity in the transmembrane domains and loops with the other PIP2;1 proteins: 91.64%, 79.23%, 86.22%, 78.75% and 76.03% sequence identity with PcPIP2;2 from *Pyrus communis*, VvPIP2 from *Vitis vinifera*, RcPIP2;1 from *Ricinus communis*, AtPIP2;1 from *Arabidopsis thaliana*, OsPIP2;1 from *Oryza sativa*, respectively (Fig 1). The MzPIP2;1 has six transmembrane domains and the signature sequences of plasma membrane water channel protein family (GGGANXXXXGY and TGI/TNPARSL/FGAAI/VI/VF/YN). MzPIP2;1 possesses the typical features of aquaporin. It has two conserved NPA domains (Asn-Pro-Ala). One is located between the second and the third transmembrane domains, and the other one is located between the fifth and the sixth transmembrane domains (Fig 1). In addition, phylogenetic tree showed that MzPIP2;1 was grouped into the PIP2 subfamily, suggesting it is a member of aquaporin PIP2 subfamily (Fig 2).

### MzPIP2;1 is localized on the plasma membrane

**Fig 3:**
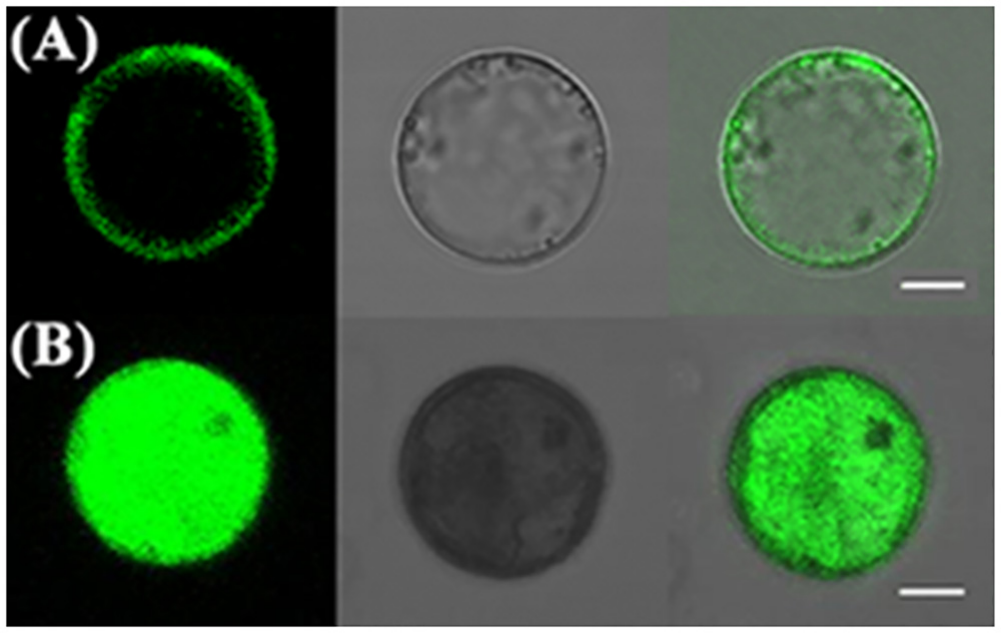
The subcellular localization of MzPIP2;1 on plasma membrane. The transgenic *Arabidopsis* protoplasts transiently expressing MzPIP2;1-eGFP fusion protein (A) and the transgenic *Arabidopsis* protoplasts transiently expressing eGFP protein (B) were observed using confocal microscopy with 600×magnification (objective magnification × eyepiece magnification = 60×10). Left: Green fluorescence images; Middle: Bright-field images; Right: Merged fluorescent images. Bar, 10μm.

The transiently transformed *Arabidopsis* protoplasts expressing *MzPIP2;1*-*eGFP* and *eGFP* were observed under confocal microscopy to confirm the subcellular localization of MzPIP2;1. Clearly, MzPIP2;1-eGFP was localized at the plasma membrane, and the control eGFP was presented in the whole protoplast (Fig 3A and 3B). These results identified that MzPIP2;1 was localized on the plasma membrane.

### The detection of *GUS* expression driven by *MzPIP2;1* promoter in transgenic *Arabidopsis* plants under normal condition

**Fig 4:**
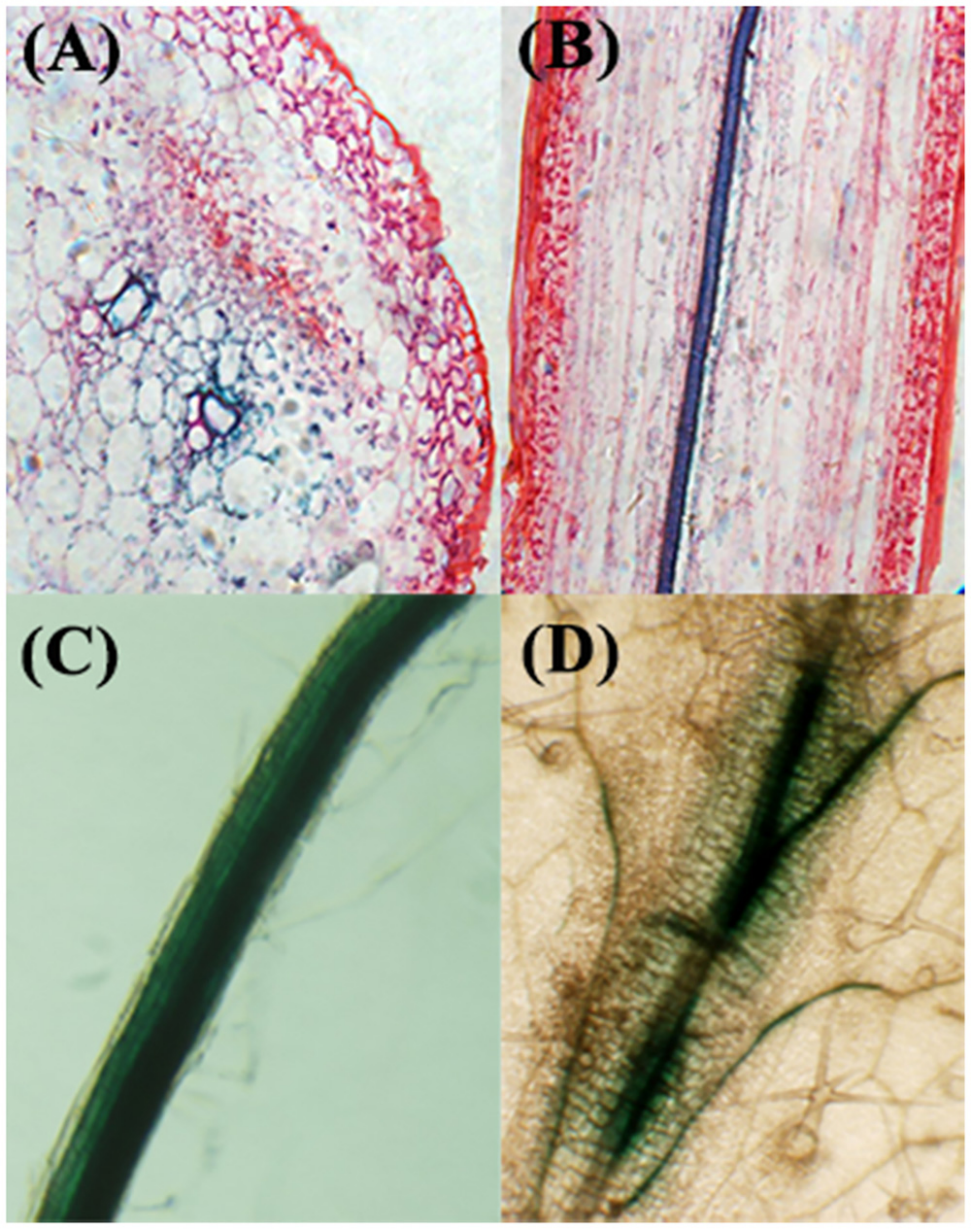
The observation of *GUS* expression driven by *MzPIP2;1* full-length promoter in transgenic *Arabidopsis* seedlings at different tissues under normal condition. The transverse section (A), longitudinal section (B) of paraffin sections in stem, root (C) and leaf (D) are observed by light microscope with 600× magnification (objective magnification × eyepiece magnification = 60×10).

In order to trace the localization of MzPIP2;1 protein, both longitudinal section and transverse section of paraffin sections in stems were stained by GUS dying. It was clear that the vascular tissues were dyed deeply, especially the parenchyma cells around vessels (Fig 4A and 4B), suggesting that the MzPIP2;1 might function in the water movement of vascular systems. In addition, the MzPIP2;1 exits in all the epidermal root cells and the inner vascular tissues, while in leaves, it is only presented in vascular tissues (Fig 4C and 4D).

### Generation of transgenic *Arabidopsis* plants ectopically expressing *MzPIP2;1*

**Fig 5:**
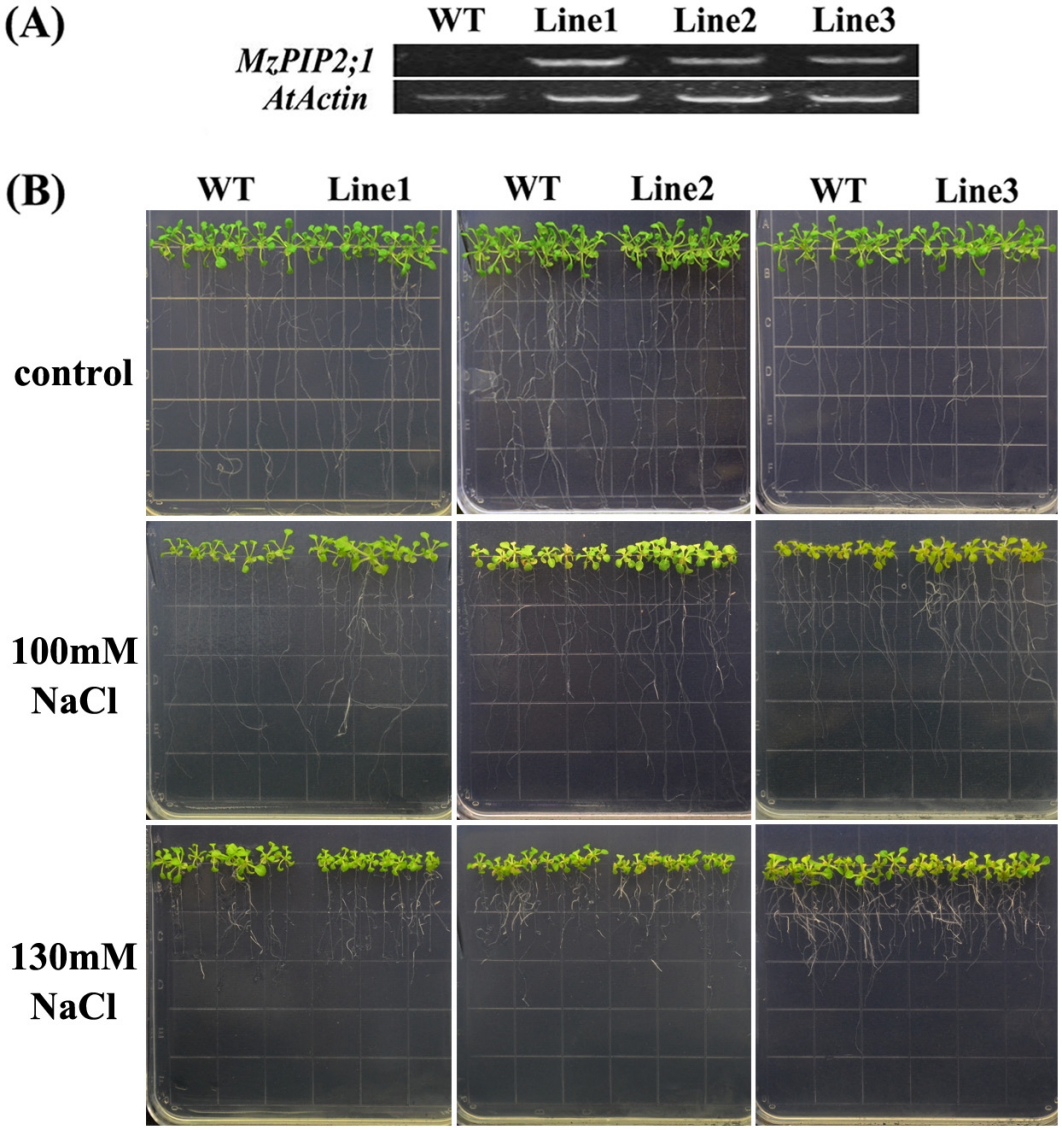
The phenotype of young transgenic *Arabidopsis* plants ectopically expressing *MzPIP2;1* under salt stress. (A) The expression levels of *MzPIP2;1* in three transgenic lines and wild-type by semi-quantitative RT-PCR, with the *AtActin* as internal gene; (B)The phenotype of three transgenic lines and wild-type under 100 NaCl and 130mM NaCl treatments. WT: wild-type.

To investigate the role of *MzPIP2;1* in the response to abiotic stresses, the T3 single-insertion homozygous *Arabidopsis* lines expressing *MzPIP2;1* driven by CMV 35S promoter were used for phenotyping. The T0 seeds were sowed in the soil and selected by spraying 0.01% Basta to obtain the T1 generation transgenic plants. A total of thirty positive T1 transgenic lines were confirmed by PCR, among which nine lines were found to be single-inserted showing the 3:1 segregation ratio of T2 plants. Then homologous single-inserted T3 plants were obtained only when no segregation happened. Finally three single insertion homologous T3 lines with high expression levels of *MzPIP2;1* were selected for phenotyping (Fig 5A).

### The young transgenic *Arabidopsis* plants ectopically expressing *MzPIP2;1* showed tolerance to slight salt stress, not to moderate salt stress

**Fig 6:**
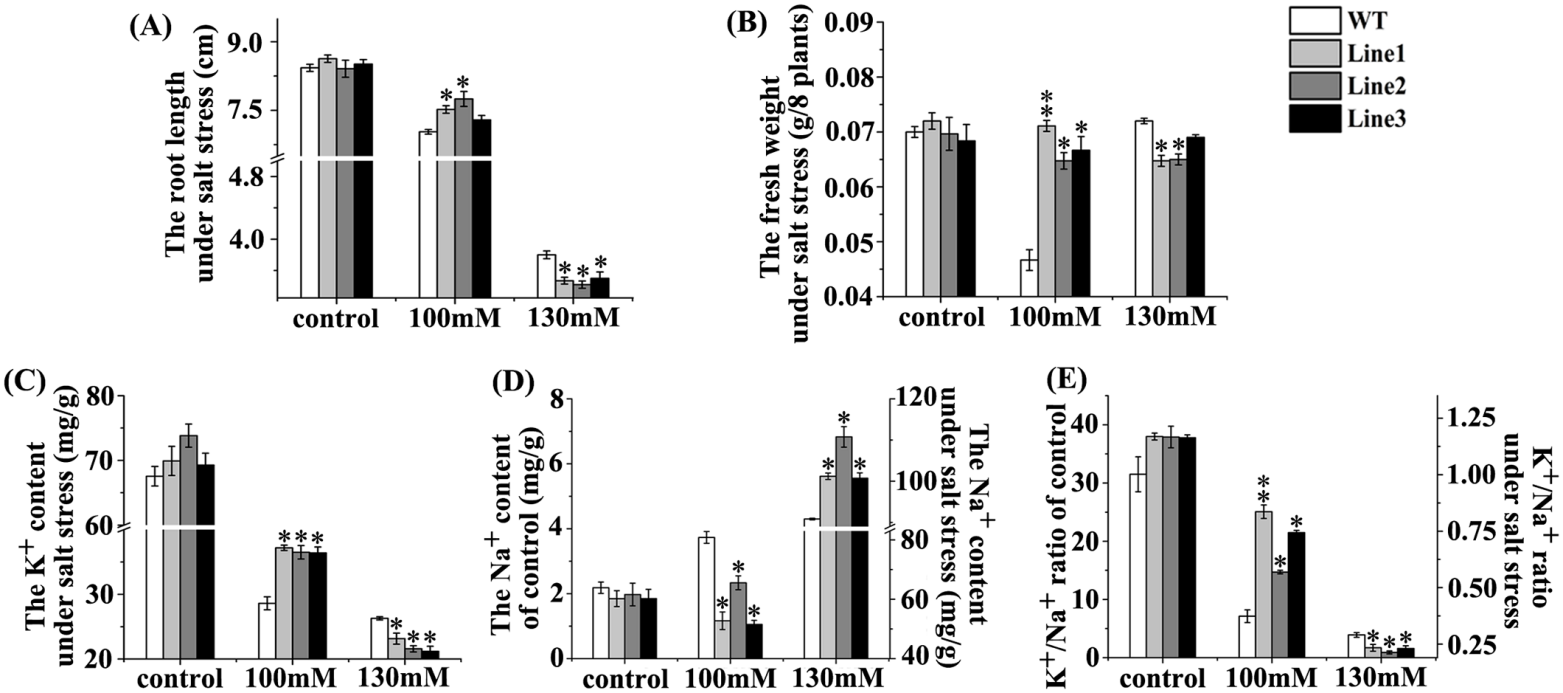
The growth characteristics of young transgenic *Arabidopsis* plants ectopically expressing *MzPIP2;1* under salt stress. The root length (A), the fresh weight (B), the K^+^ content (C), the Na^+^ content (D) and the K^+^/Na^+^ ratio (E) of three transgenic lines and wild-type control under 100 NaCl and 130mM NaCl treatments. WT: wild-type.

All of the three T3 generation single-insertion homozygous transgenic lines were tolerant to 100mM NaCl. The transgenic plants had longer root length and heavier fresh weight than wild-type (Figs 5B, 6A and 6B). However, when the NaCl level rose up to 130mM, compared with wild-type, the up-ground parts of all the three transgenic lines showed weaker growth, shorter root lengths and lighter fresh weight (Figs 5B, 6A and 6B). Accordingly, the transgenic lines had higher K^+^ content and lower Na^+^ content than wild-type under slight salt stress of 100mM NaCl (Fig 6C and 6D). While under moderate salt stress of 130mM NaCl, dramatically reverse results happened. In all the three transgenic lines, the K^+^ contents were lower and Na^+^ contents were higher than the wild-type (Fig 6C and 6D). Therefore, the K^+^/Na^+^ ratios of the transgenic plants under 100mM NaCl treatment were higher than wild-type, and lower under 130mM NaCl (Fig 6E). Taken together, these young transgenic plants ectopically expressing *MzPIP2;1* were tolerance to slight salt stress, but sensitive to moderate salt stress.

### *MzPIP2;1* conferred the transgenic *Arabidopsis* plants enhanced drought tolerance

**Fig 7:**
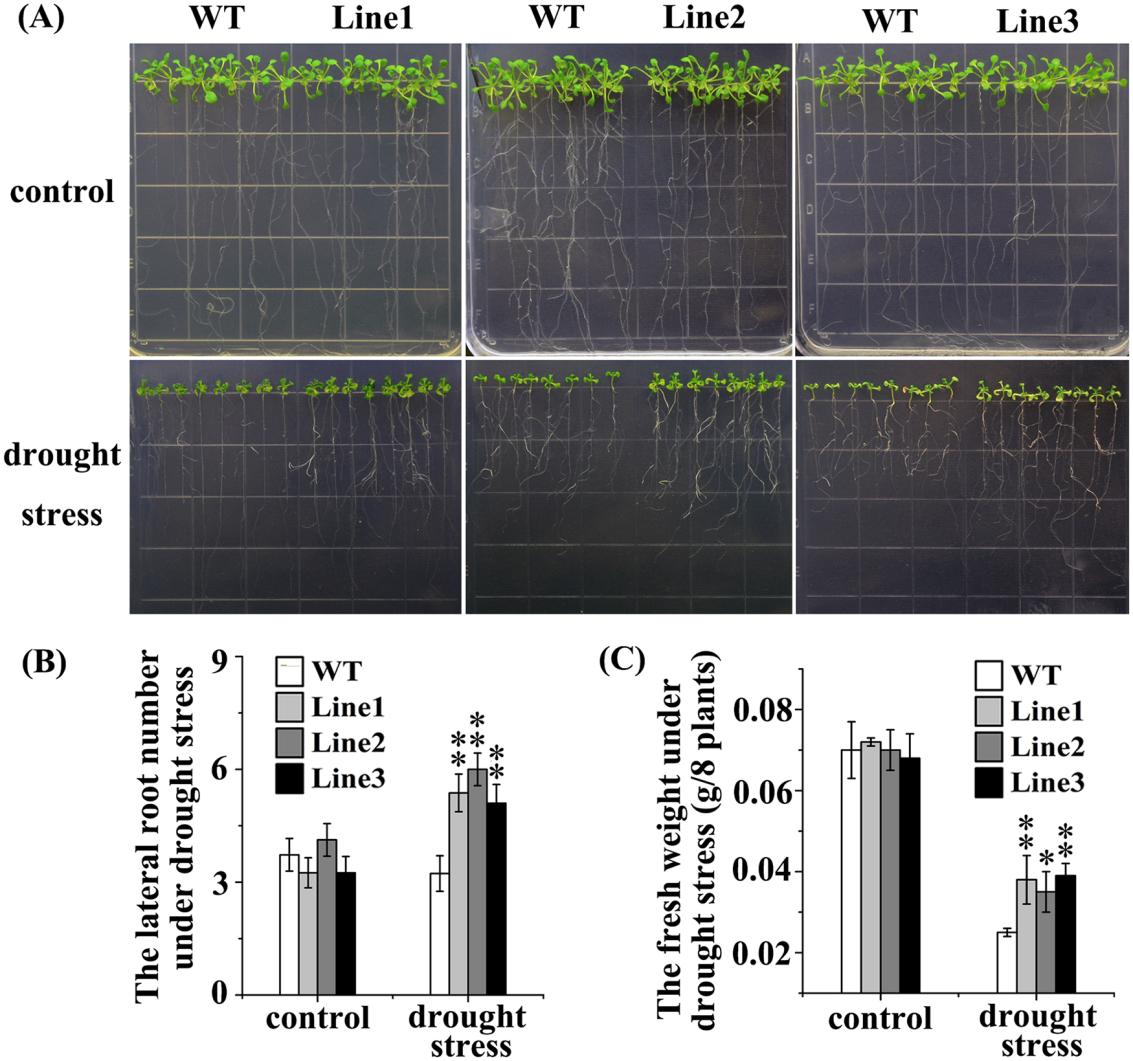
The phenotype of young transgenic *Arabidopsis* plants ectopically expressing *MzPIP2;1* under drought stress. The growth condition (A), the lateral root number (B) and the fresh weight (C) of three transgenic lines and wild-type control. WT: wild-type.

Under drought stress, all the three transgenic lines grew better than wild-type. They had more lateral roots and higher fresh weight than wild-type under drought stress (Fig 7), suggesting that the ectopically expressed *MzPIP2;1* conferred drought tolerance to the transgenic seedlings. But there wasn’t significant difference between transgenic and wild-type seedlings under cold stress.

### The *GUS* expression driven by *MzPIP2;1* promoters in young and adult transgenic *Arabidopsis* plants under abiotic stresses

**Fig 8:**
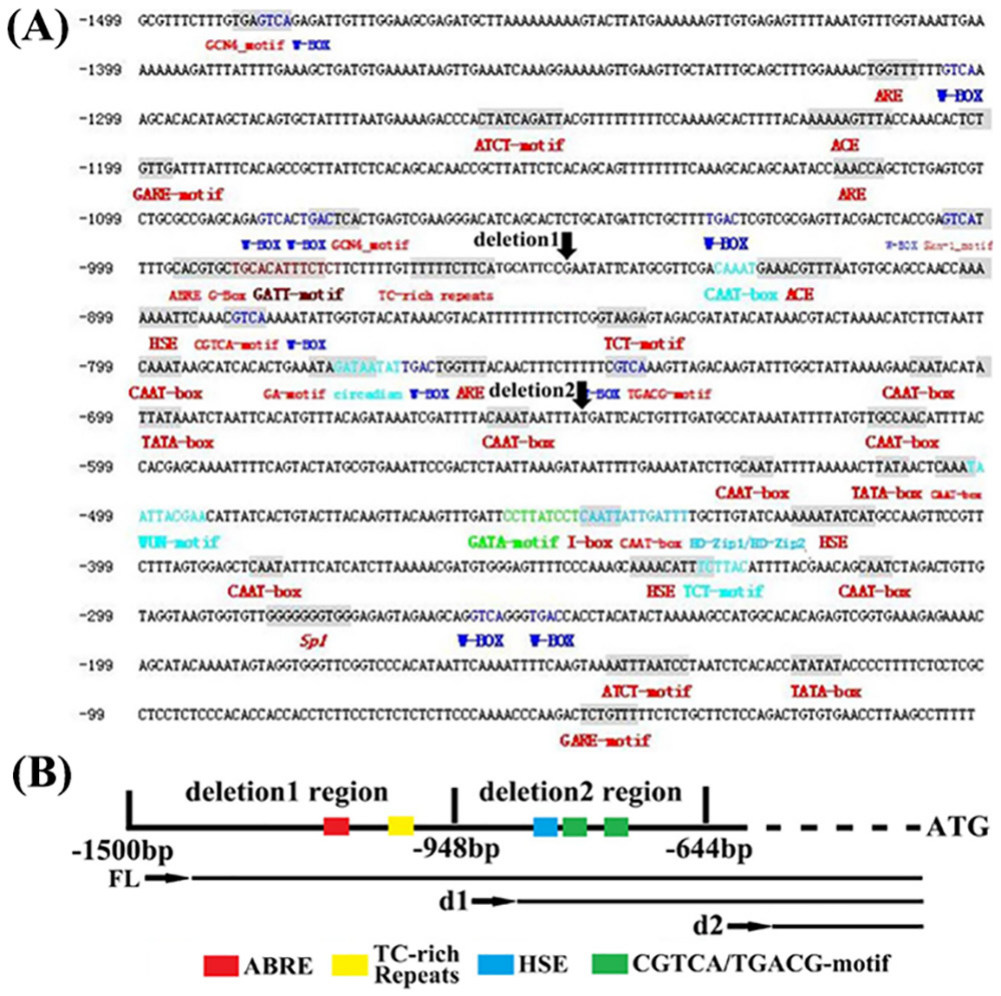
The *cis*-elements analysis and diagram of *MzPIP2;1* promoter deletion. (A) the *cis*-elements analysis; (B) the diagram. The *cis*-elements analysis is conducted at http://bioinformatics.psb.ugent.be/webtools/plantcare/html/. FL: full-length promoter; d1:deletion1; d2: deleion2.

To determine the key *cis*-elements in response to the abiotic stresses, the promoter deletion experiment was conducted. The expression of reporter gene *GUS* driven by different promoter was detected (Fig 8A). The deletion1 region was from -1499bp to -948bp, and the deletion 2 region was from -948bp to -644bp (Fig 8B).

**Fig 9:**
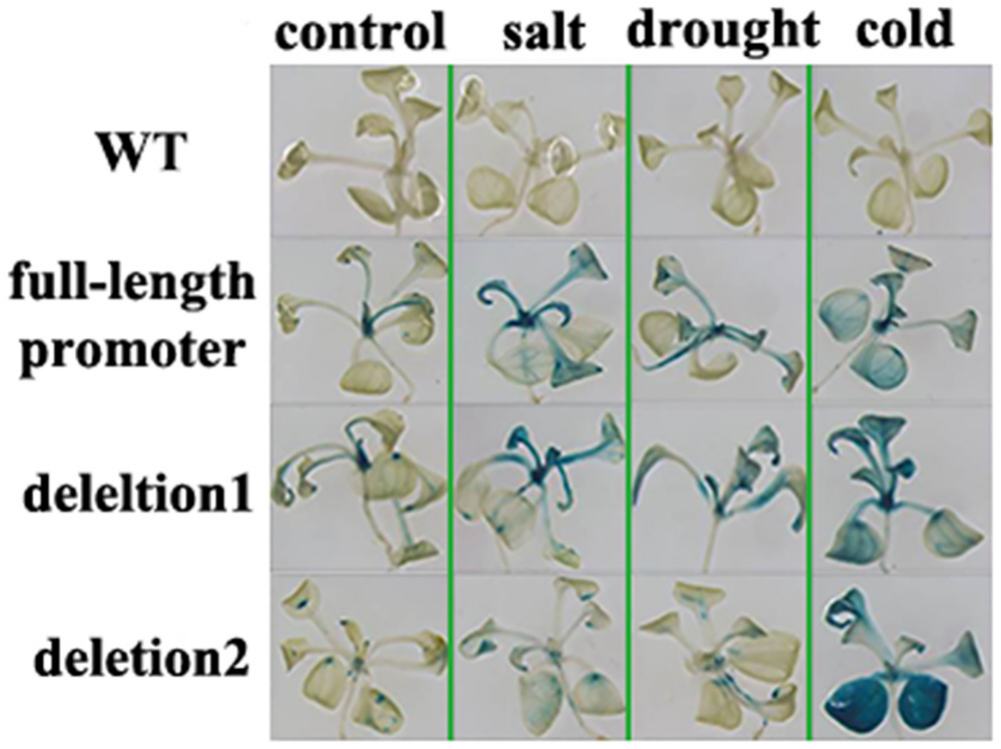
The expression of *GUS* driven by *MzPIP2;1* promoters in young seedlings of transgenic *Arabidopsis* under abiotic stresses. The up-ground parts are observed by stereoscope with 7.3× magnification (objective magnification × eyepiece magnification = 0.73×10). WT: wild-type.

Under salt stress, the *GUS* expression driven by *MzPIP2;1* full-length promoter and two deletions were detected in stems and leaves under different stresses. Compared with the control, the *GUS* expression was induced when driven by the full-length promoter and deletion1 under salt stress. But *GUS* expression was not inducible when driven by deletion2 (Fig 9). Under drought stress, similar expression pattern was observed. The full-length promoter and deletion1 responded to drought stress similarly, while deletion2 was not able to respond any more (Fig 9). Under cold stress, the full-length promoter and deletion1 positively responded to cold stress similarly, while deletion2 showed dramatically enhanced response (Fig 9), suggesting a *cis*-element bound by a repressor in this region. It inferred the key *cis*-element might exist in the deletion 2 region in the cold response of young seedlings.

**Fig 10:**
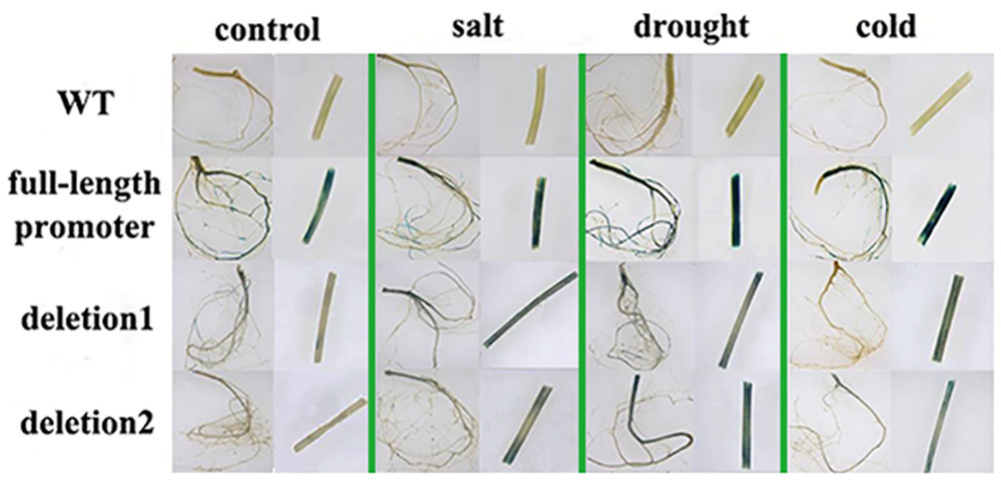
The expression of *GUS* driven by *MzPIP2;1* promoters in adult seedlings of transgenic *Arabidopsis* under abiotic stresses. The roots and stems are observed by stereoscope with 7.3× magnification (objective magnification × eyepiece magnification = 0.73×10). WT: wild-type.

In adult transgenic seedlings, the *GUS* expression was observed in the roots and stems. Compared with the observation in young seedlings, there was no significant difference in *GUS* staining between deletion1 and deletion2. The strongest staining happened in the full-length promoter (Fig 10), suggesting the key *cis*-element for stress response might exist in the deletion1 region in adult period.

## Discussion

In the water uptake and transportation, many aquaporin proteins localized at different tissues, play important roles in conducting various transmembrane water movements. In this study, we targeted at the one involved in the key water transmembrane transportation paths, by which water entered root epidermal cells and went into parenchyma cells. Because this decides the efficiency of water uptake and usage, it might play a critical role in response to water deficit.

Our results showed that the expressions of four *PIP* candidate genes from a salt-tolerant apple rootstock *Malus zumi* Mats were induced by salt stress (S1 Fig). The bioinformatics research showed all the possible PIPs have the signature domains of AQP (GGGANXXXXGY and TGI/TNPARSL/FGAAI/VI/VF/YN), which belong to the plasma membrane water channel protein family. They all have two NPA (Asn-Pro-Ala) conserved domains forming the hydrophilic channel of aquaporins (S2 Fig). In order to isolate our target *PIP* gene, the *GUS* gene driven by these *PIP* promoters were stably transformed and expressed in *Arabidopsis*, respectively. The *GUS* gene driven by *MzPIP2;1* promoter was expressed in both root epidermal cells, parenchyma cells around stem xylem vessels and vascular tissues of leaves. Its localization suggested MzPIP2;1 is possibly our target protein involved in water uptake and radical flow. In previous studies, many aquaporins were found localized in vascular bundles and adjacent tissues of stems or leaves, and some were also expressed in roots [38–41]. But there wasn’t a report of any plasma membrane aquaporins in charge of both water uptake and transportation. For the first time, we found that *MzPIP2;1* was expressed specifically in root epidermal cells, vascular tissues of stems and leaves, implying its important roles in both water uptake and usage of the whole plant.

If MzPIP2;1 could improve the water uptake and usage efficiency as expected, the ectopically expressed *MzPIP2;1* could confer transgenic plants enhanced tolerance to water deficit. Therefore, we observed the phenotype of transgenic *Arabidopsis* under salt and drought stresses. The transgenic plants were more tolerant to drought stress. Their fresh weight and lateral root number were significantly higher than wild-type (Fig 7). Under slight salt stress, the transgenic plants were more tolerant than the wild-type (Fig 5). Obviously, the increased *MzPIP2;1* expression resulted in more efficient water uptake and radial transportation, which increased the tolerance of transgenic plants to drought and slight salt stresses. But out of our expectation, the transgenic plants were even more sensitive to moderate salt stress (Fig 5). Compared with drought stress, salt stress causes not only water deficit, but also ionic toxicity. When the Na^+^ accumulates to a high level or K^+^/Na^+^ ratio decreases, the ionic toxicity happens. It could destroy integrity of cellular membranes, inhibit the activities of various enzymes and affect nutrient acquisition and function of photosynthetic apparatus [42]. In order to find if the ionic toxicity happened to transgenic plants under moderate salt stress, the Na^+^ and the K^+^ contents were detected in transgenic plants under salt stress in different degrees. In the transgenic plants under slight salt stress, the K^+^/Na^+^ ratio was higher than wild-type, but it was significantly lower than wild-type under moderate salt stress (Fig 6E). This dramatic decrease of K^+^/Na^+^ ratio resulted from both the increase of Na^+^ level and decrease of K^+^ level. It was reported that there is a possible trafficking competition between PIP protein and the SYP121 (a soluble N-ethylmaleimide-sensitive factor protein attachment protein receptor, also a plasma membrane resident syntaxin), involved in trafficking of K^+^ channels [4, 43]. Therefore, the increased PIP protein in transgenic plants might reduce the abundance of K^+^ channels in membranes by trafficking competition and decrease the K^+^ absorbance. In our research, significant decrease of K^+^ absorbance happened in transgenic plants. It might result from both of the well-known Na^+^ /K^+^ channels competition and the possible trafficking competition between K^+^ channels and PIPs. When transgenic plants were subjected to moderate salt stress, the abundant PIP proteins increased plant tolerance, but the ionic toxicity made the plants less resistant to salt stress [44, 45]. The sensitivity to moderate salt stress mainly resulted from the extremely accumulated sodium. In addition, the post-translational modifications including phosphorylation and heteromerization might also partially explain this sensitivity to moderate salt stress. Under higher salt stress, more gates for water movements into roots were required. Although the *MzPIP2;1* gene was highly expressed, the kinase for the its phosphorylation and other PIPs for heteromerization were not expressed in high level accordingly. This may limit activity of over-produced MzPIP2;1.

Although aquaporins have been intensively researched, the upstream genes regulating their expression remain unknown. In this research, the promoter deletion experiment was performed to find the key *cis*-elements involved in abiotic stress response for further research. In the young seedlings, the result suggested that the key *cis*-elements might be in deletion2 region. In addition, it was very interesting that the *GUS* expression driven by deletion2 was induced dramatically by cold stress in young seedlings, compared with the full-length promoter and deletion1 (Fig 9), suggesting the key *cis*-element bound by a repressor was in the deletion2 region. In this region, there are several candidate cis-elements (Fig 8B). The HSE is recognized and bound by heat shock factors (HSFs) to induce gene expression of heat shock proteins (HSPs). Abundant HSPs could relieve plants from drought and salt stresses [46–48]. Furthermore, there were two jasmonic acid methylester (MeJA)-responsive elements in this region (Fig 8B) [49,50]. MeJA was also a signal involved in the abiotic stress response [51]. Therefore, it was possible that the HSE and the MeJA-responsive elements played a key role in stress response by interaction with upstream transcription factors. But there were not any reports of cold responsive repressors interacting with these elements. The possibility could not be excluded that an unknown cis-element was involved in the cold response.

Because the young seedlings and the adult plants were in the different developmental stages, it was very possible that the different transcription factors and partner combination or different activity of these proteins were presented. Therefore, the *GUS* expression pattern driven by full-length promoter and different deletions of *MzPIP2;1* were also detected in the adult period under salt, drought and cold stresses. Different expression pattern were observed in the adult plants, compared with young plants. It suggested that the key elements on responding stresses in adult plants might be in deletion1 region. There were some well-known stress-responsive elements in this region including ABRE motif and a TC-rich repeats (Fig 8B). The ABRE (ACGTGG/TC) is a conserved ABA-responsive *cis*-element [52], existing in the promoters of many genes regulated by stress hormone ABA [53]. But multiple ABREs were needed for the ABRE-binding proteins [54–56]. Therefore, it was possible that the single ABRE in the promoter was not functional. In addition, the TC-rich element was also a stress response element [57, 58]. It might play a crucial role in stress response of adult period, instead of young period, which led to the different stress response patterns between the young and the adult periods.

## Conclusion

In conclusion, MzPIP2;1, a PIP2 aquaporins subgroup member, plays a crucial role in the radial water movement in water uptake from soil to root epidermal cells and transportation from vessels to parenchyma cells in the whole plant. Therefore, the ectopically expressed *MzPIP2;1* altered the salt and drought tolerance of transgenic *Arabidopsis* plants. In addition, the *cis*-elements in response to salt, drought and cold stresses were isolated, which paved a way to further isolate the upstream genes regulating the MzPIP2;1 expression.

## Supporting Information

S1 Fig The expression of four candidate *MzPIPs* in roots and leaves of *Malus zumi* Mats under salt treatment for different time. The *Malus zumi* Mats seedlings were treated with 150mM NaCl under normal growth condition (16/8h light/dark cycle (~150 μEm^-2^ sec^-1^) at 22±2°C) and sampled at 0, 4, 8, 12, 24h, 3 and 5days, respectively. The *MzACTIN* gene is as control.
(TIF)

S2 Fig The amino acid sequences of the other three candidate MzPIPs. MzPIP1;1 (A), MzPIP1;2 (B) and MzPIP2;2 (C). The transmembrane domains were underlined; NPA box was paned; Asterisk, the sequences of PIPs were dashed; the stop coden was labeled with *.
(TIF)
