# Supplementary figures and images for: MzPIP2;1: An Aquaporin Involved in Radial Water Movement in Both Water Uptake and Transportation, Altered the Drought and Salt Tolerance of Transgenic Arabidopsis
Source: PLoS One. 2015 Nov 12;10(11):e0142446. doi: 10.1371/journal.pone.0142446 (PMC4643029; doi:10.1371/journal.pone.0142446)

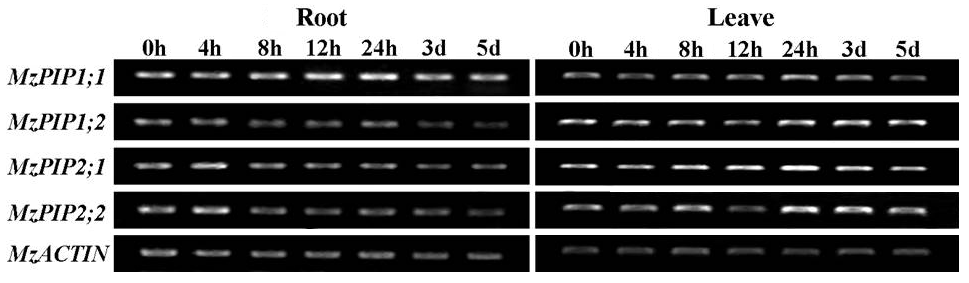

Supplement: S1 Fig — The Malus zumi Mats seedlings were treated with 150mM NaCl under normal growth condition (16/8h light/dark cycle (~150 μEm-2 sec-1) at 22±2°C) and sampled at 0, 4, 8, 12, 24h, 3 and 5days, respectively. The MzACTIN gene is as control. (TIF) [file pone.0142446.s001.tif]

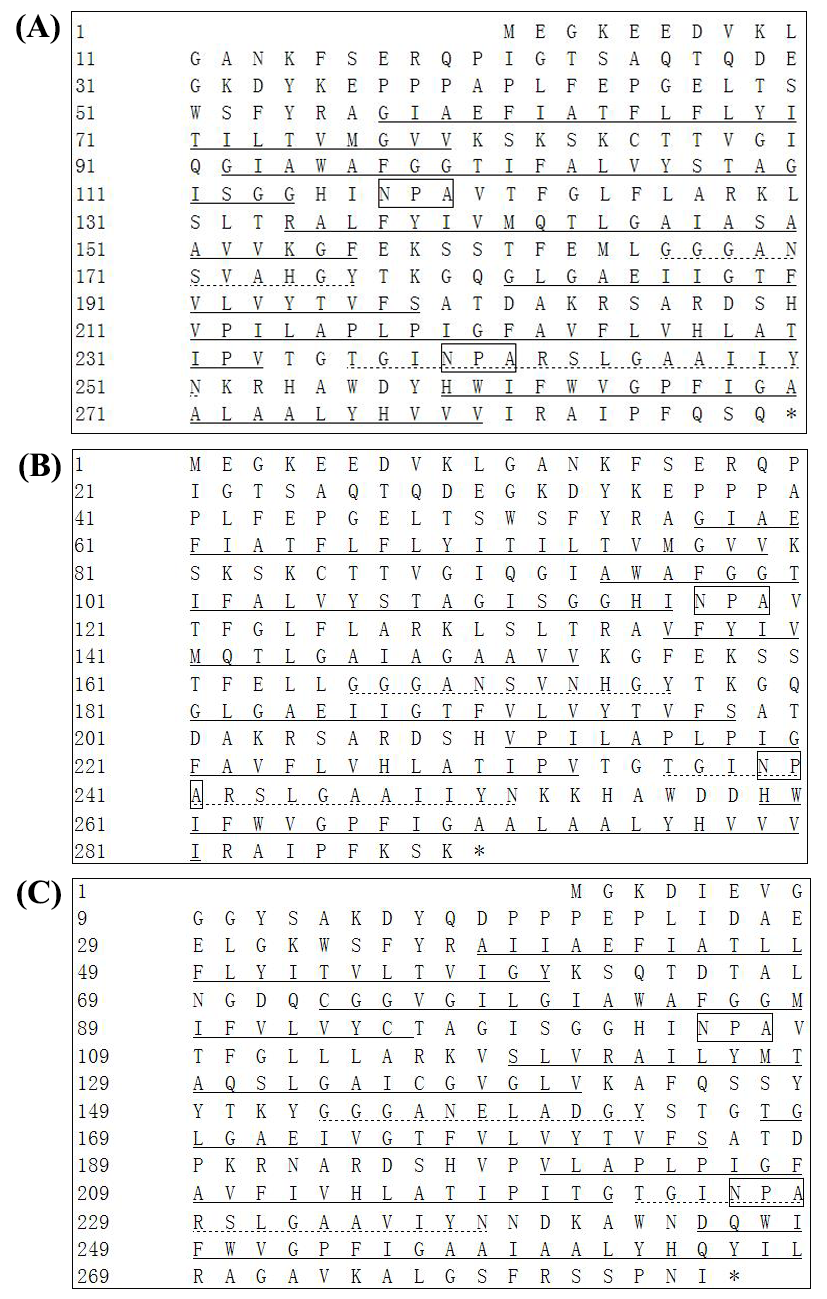

Supplement: S2 Fig — MzPIP1;1 (A), MzPIP1;2 (B) and MzPIP2;2 (C). The transmembrane domains were underlined; NPA box was paned; Asterisk, the sequences of PIPs were dashed; the stop coden was labeled with *. (TIF) [file pone.0142446.s002.tif]
